# Severe Hyperkalemia in a Child with Vomiting and Diarrhea

**DOI:** 10.5811/cpcem.21173

**Published:** 2024-11-09

**Authors:** Abdullah Khan

**Affiliations:** Sidra Medicine, Department of Emergency Medicine, Ar-Rayyan, Qatar

**Keywords:** hyperkalemia, congenital adrenal insufficiency, ECG changes in hyperkalemia

## Abstract

**Case Presentation:**

A 13-month-old child with past medical history of congenital adrenal insufficiency presented to the emergency department with vomiting and diarrhea. Initially the child was noticed to have bradycardia with normal blood pressure. An electrocardiogram (ECG) showed tall T waves, broad QRS complex, and widened PR interval suggestive of severe hyperkalemia. The initial blood gas showed potassium of 10.7 millimoles per liter. The patient was started on calcium gluconate with immediate resolution of ECG changes. Further management with insulin, dextrose, and sodium polystyrene sulfonate led to normal potassium levels.

**Discussion:**

Hyperkalemia is a life-threatening condition in children, especially in those with congenital adrenal insufficiency. The ECG showed different changes as the levels of serum potassium levels increased ranging from tall T waves, wide QRS complex, increased PR interval to arrythmias. Immediate treatment with calcium gluconate in such cases has significant cardioprotective effect. It is important to recognize the ECG changes manifested by changes in serum potassium levels. Our patient had classic ECG changes manifested in severe hyperkalemia.

## CASE PRESENTATION

A 13-month-old male child with a previous history of congenital adrenal insufficiency presented to the emergency department with multiple episodes of vomiting (non-bloody and non-bilious) and diarrhea (non-bloody and non-mucoid). The patient was not able to take his regular hydrocortisone doses at home. Initially the child did not look well and had bradycardia (heart rate 52–59 beats per minute) with normal blood pressure. The patient was attached to a cardiac monitor and a wide QRS-complex rhythm was noticed.

**Image 1:**
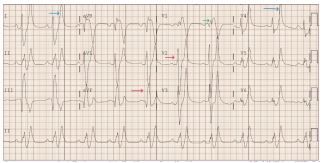
Electrocardiogram suggesting signs of hyperkalemia. Lead V_1_ shows rSr’ (green arrow), Precordial and limb leads show wide QRS complex (red arrows) and tall T waves (blue arrows).

An electrocardiogram (ECG) was obtained. The ECG showed sinus bradycardia with regular rhythm. The QRS axis was rightward (106 degrees), which is normal in this age group. There were tall, peaked upright T waves in all precordial and limb leads, except for V_1_ where the T wave was deeply inverted. The QRS complexes were wide at 196 milliseconds (ms) (normal range 54–88 ms). The PR interval was prolonged at 226 ms (86–151 ms). There was rSr’ pattern in the V_1_ lead with widening of the S wave in lead I and aVF, suggesting right bundle branch block (Image 1).

The initial venous blood gas results showed potassium of 10.7 millmoles per liter (mmol/L) (reference range 3.5–5.2 mmol/L) and sodium of 122 mmol/L (135–145 mmol/L). The initial complete metabolic panel showed a blood urea nitrogen of 18.1 mmol/L (3.2 to 7.9 mmol/L) and creatinine of 132 mmol/L (13–35 mmol/L). With ECG findings suggestive of severe hyperkalemia, albuterol and calcium gluconate (100 milligrams per kilogram [mg/kg]) were started. The patient developed an episode of ventricular tachycardia (heart rate 200–215) with normal blood pressure. Albuterol was stopped, and calcium gluconate was continued. The patient successfully reverted to normal sinus rhythm with calcium gluconate (Image 2). A stress dose of hydrocortisone (25 mg) was given, and insulin (0.1 units/kg/hour) with dextrose 10% infusion was started. A 20 millimeter (mL) per kg bolus of normal saline was also given due to acute kidney injury. A small dose of furosemide (0.5 mg/kg) and rectal sodium polystyrene sulfonate (1 gram/kg) were also administered. The potassium levels were corrected over a period of five hours. The patient was observed in pediatric intensive care unit for 24 hours and discharged without any complications.

## DISCUSSION

The ECG demonstrated typical changes of severe hyperkalemia. Hyperkalemia is a life-threatening condition that if not treated can lead to arrhythmias and asystole. Hyperkalemia produces a variety of dose-dependent changes on ECG that include tall peaked T waves (5.5–6.5 mmol/L); prolongation of the PR interval and QRS widening (6.5–7.5 mmol/L); ST-segment changes, QTc prolongation, and decrease in amplitude of P wave (7–8 mmol/L); and marked widening of QRS and sine wave pattern and arrhythmias. 1 In children the abnormal values of different waves and segments of ECG are age and sex dependent, and upper limits of normal values are described as the 98^th^ percentile. The 98^th^ percentile values for male children between 1–3 years of age for PR-interval QRS duration and QTc-interval are 151 ms, 88 ms, and 455 ms, respectively. 2 The ECG in our patient demonstrated prolongation of all these segments.

**CPC-EM Capsule**

What do we already know about this clinical entity?

*Severe hyperkalemia can be by acute illness in children with congenital adrenal insufficiency.*

What is the major impact of the image(s)?

*It is important to identify electrocardiographic changes associated with hyperkalemia.*

How might this improve emergency medicine practice?

*In children with congenital adrenal insufficiency presenting with hyperkalemia triggered by acute illness, stress doses of hydrocortisone must be administered.*

Hyperkalemia in children is usually caused by impaired excretion of potassium (adrenal or renal insufficiency), and movement of intracellular potassium to the extracellular space (acidosis, diabetes, tumor lysis syndrome, rhabdomyolysis), as well as drugs and toxins (spironolactone, angiotensin-converting enzyme inhibitors, beta blockers and calcium channel blockers). 3 If ECG changes are present, the immediate step of administering cardiac membrane-stabilizing agent such as calcium gluconate 10% followed by potassium lowering agents, such as albuterol, insulin and dextrose, furosemide and sodium polystyrene sulfonate, is warranted. 1,3 In our patient, hyperkalemia was caused by the underlying adrenal insufficiency exacerbated by missed doses of hydrocortisone due to vomiting and acute illness.

Adrenal insufficiency is caused by dysfunction at any level of hypothalamic-pituitary-adrenal axis. Hence, it can be broadly classified as primary (dysfunction of adrenal gland), secondary (decreased release of adrenocorticotropic hormone from the pituitary gland), and tertiary (decreased release of corticotropin hormone from the hypothalamus). 4 Adrenal insufficiency leads to decreased production of glucocorticoid (cortisol) and mineralocorticoid (aldosterone). Aldosterone regulates sodium absorption and potassium excretion at the level of distal tubules of nephrons; hence, its deficiency leads to hyponatremia and hyperkalemia. Cortisol regulates serum glucose levels and blood pressure in periods of stress. Patients with adrenal insufficiency develop adrenal crises in acute illness. Therefore, in addition to management of hyperkalemia it is important to administer stress doses of hydrocortisone (25 mg for ages 0–3 years) to achieve both glucocorticoid and mineralocorticoid effect in cases of congenital adrenal insufficiency.^5^

## Figures and Tables

**Image:**
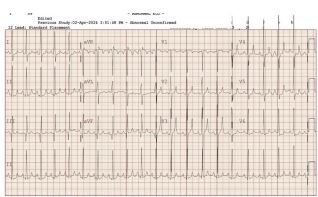
Normal sinus rhythm after administration of calcium gluconate in a pediatric patient with hyperkalemia.
